# Job Satisfaction of Registered Respiratory Therapists in Primary Care: Addressing Recruitment and Retention in Ontario and Manitoba †

**DOI:** 10.3390/bs15101301

**Published:** 2025-09-24

**Authors:** Sandra Biesheuvel, Dayajyot Kaur, Song Lee Han, Olsen Jarvis, Louise Chartrand

**Affiliations:** 1 Department of Respiratory Therapy, College of Rehabilitation Sciences, University of Manitoba, Winnipeg, MB R3T 2N2, Canada; sandra.biesheuvel@umanitoba.ca (S.B.); dayajyot.kaur@gmail.com (D.K.); jarviso2@myumanitoba.ca (O.J.); 2 Best Care, Windsor, ON N8W 5V7, Canada; shan044@michener.ca

**Keywords:** job satisfaction, primary care, Mottaz framework, respiratory therapist, allied healthcare professional, Canada, recruitment, retention

## Abstract

Registered Respiratory Therapists (RRTs) have unique skills in managing chronic obstructive pulmonary disease (COPD) in primary care settings. With an 82% increase in COPD diagnoses between 2000 and 2010 in Canada, the fact that over 10% of Canadians aged 35 and older are living with COPD, and primary care reform in Ontario and Manitoba, we would expect an increasing number of RRTs working in this setting. However, this is not happening. Through the concept of job satisfaction, we want to investigate the barriers of integrating this allied healthcare professional into primary care settings. Using a pragmatic approach, we examined RRT job satisfaction in primary care via semi-structured interviews of 19 RRTs in Manitoba and Ontario in 2018 and 2019. A combined inductive and deductive (Mottaz framework) analysis approach allowed us to cross reference work context with job satisfaction. The context in which primary care is operationalized impacts RRT job satisfaction. In Ontario, retention of RRTs in primary care was the main issue due to lower salaries and benefits. In Manitoba, recruitment of RRTs in primary care was the main issue due to lack of human resources and funding. Efforts should be made to address gaps in job satisfaction of RRTs in primary care. To improve retention in Ontario, RRTs should be compensated similarly to their counterparts in acute care. In Manitoba, there should be increased positions for RRTs in primary care. Developing strategies for enhancing job satisfaction will ensure the delivery of high-quality, patient-centered care. This study provides both theoretical and practical contributions to primary care workforce research. Theoretically, our findings demonstrate how contextual factors moderate job satisfaction, showing that the primary care context produces various work situations and heavily impacts work satisfaction. Practically, our results offer specific guidance for healthcare policymakers and administrators, including standardizing compensation across care settings, converting part-time positions to full-time roles, and developing targeted educational initiatives to improve workforce recruitment and retention in underserved areas.

## 1. Introduction

Registered Respiratory Therapists (RRTs) are common throughout Canada, with 46,700 employed in 2021, predominantly in hospitals in acute care settings such as emergency rooms (ERs) and intensive care units (ICUs) (Government of Canada, 2023). Their scope of practice and specialized knowledge of the cardiorespiratory system make RRTs ideal candidates for managing chronic respiratory disorders, providing smoking cessation, and educating patients on respiratory health (Slack et al., 2018). In Canada, the demand for RRTs in primary care has risen because of the increased prevalence of chronic lung disease (GOLD, 2018). According to the Global Initiative for Chronic Obstructive Lung Disease (GOLD), primary care settings are best suited to manage chronic lung disorders (GOLD, 2018).

In Canada, the demand for specific types of healthcare professionals depends on government spending and patients’ needs, while supply depends on recruitment and retention (T. Lee et al., 2019). Healthcare in Canada is the dual responsibility of federal and provincial governments where funds are transferred to the provinces to develop and manage healthcare services (S. K. Lee et al., 2021). Recognizing the importance of primary care for improving chronic disease management and overall delivery of health services; in the early 2000s, the federal government reached an agreement with provincial and territorial leaders to reform their healthcare systems (Thompson, 2020). A key feature was providing multidisciplinary health services. Funds were made available for allied healthcare professionals (AHPs) such as RRTs to team with primary care physicians in caring for patients in the community across the continuum of health (Thompson, 2020).

COPD diagnoses in Canada increased by 82% between 2000 and 2010, with over 10% of Canadians aged 35 and older living with COPD by 2010 (HealthCanada, 2018). Estimated prevalence from 2019 to 2021 was 11.2–16.2%; therefore, more than 2 million Canadians live with COPD (Amegadzie et al., 2025; Leung et al., 2021). With primary care reform occurring during this same period, we would expect to see more RRTs working in this area. However, according to the College of Respiratory Therapists of Ontario (CRTO), only 2% of RRTs in Ontario are employed to manage chronic lung disease in primary care; this percentage drops to 1% in Manitoba. This disconnect between expected workforce integration and actual employment patterns represents a significant gap in our understanding of primary care workforce dynamics.

### 1.1. Research Purpose and Objectives

This study aims to investigate the barriers to RRT integration in primary care through systematic examination of job satisfaction factors. Our specific objectives are to (1) explore RRT experiences across different primary care contexts in Ontario and Manitoba, (2) identify specific factors that influence job satisfaction among primary care RRTs, and (3) develop evidence-based recommendations for improving recruitment and retention of RRTs in primary care settings.

### 1.2. Novelty and Contribution

This research makes several original contributions to the literature. First, it represents the first qualitative examination of RRT job satisfaction specifically within primary care environments. Second, through our inductive analysis, we discovered that primary care operates through distinct organizational models that fundamentally shape professional experiences, a finding that had not been previously recognized in allied health workforce research. Third, our comparative approach across two Canadian provinces with different healthcare structures reveals how provincial context influences the relationship between job satisfaction frameworks and workforce outcomes. Finally, our application of Mottaz’s job satisfaction framework to primary care RRTs provides new theoretical insights into how established frameworks perform in emerging healthcare delivery models.

### 1.3. Structure of the Article

This manuscript is organized as follows: a comprehensive literature review examines existing knowledge on job satisfaction with a focus on theory that has been used to understand it; the methodology section details our pragmatic qualitative approach using semi-structured interviews and mixed inductive-deductive analysis; results are presented by the distinct primary care models we discovered, with job satisfaction factors analyzed within each context; the discussion synthesizes findings across provinces and models while positioning results within broader healthcare workforce theory; and practical recommendations provide specific, actionable guidance for policymakers and healthcare administrators seeking to improve RRT integration in primary care.

By highlighting the barriers of recruiting and retaining RRTs in primary care in Canada, this article not only addresses a gap in the literature, but it will also suggest avenues for future research with the aim of improving job satisfaction among AHPs.

## 2. Literature Review

Job satisfaction remains a pivotal construct in organizational research, directly influencing recruitment, retention, and overall organizational effectiveness in healthcare settings. From a behavioral science perspective, understanding the theoretical underpinnings of job satisfaction is essential for developing actionable strategies that enhance employee engagement and reduce turnover among healthcare professionals. Job satisfaction in healthcare has a great impact as it affects quality, productivity, effectiveness, and healthcare costs (Karaferis et al., 2022).

Recent literature indicates a shortage of all types of allied health professionals (AHPs) worldwide (Roth et al., 2024), even though their services are known to improve health outcomes while decreasing medical expenditures (Buchan, 2010). Job satisfaction is a key component of recruitment and retention and can impact these shortages (Rad & De Moraes, 2009; Vitali et al., 2020). Satisfied employees are more productive, provide better services, and are more committed to the organizations within which they work than dissatisfied employees. By contrast, job dissatisfaction is linked to burnout and emotional distress, which can lead to higher absenteeism and turnover (Mundt & Zakletskaia, 2019). Job dissatisfaction in the healthcare industry is also linked to poor patient care (Friedberg, 2013). Examining levels of job satisfaction among respiratory therapists should therefore help us understand why so few work in primary care settings.

The two job satisfaction frameworks most often used in healthcare research, particularly for the rehabilitation professions, are Herzberg’s Motivation-Hygiene Theory and Mottaz’s work value and rewards theory (Tran et al., 2008). The Mottaz framework provides greater insight on actionable problems. This review synthesizes key theoretical frameworks and provides rationale for the adoption of the Mottaz framework as the guiding model for understanding respiratory therapist job satisfaction in primary care.

### 2.1. Maslow’s Hierarchy of Needs

Maslow’s theory posits that individuals are motivated by a hierarchy of needs, ranging from physiological and safety needs to belonging, esteem, and self-actualization. In healthcare workplace contexts, satisfaction is achieved as employees’ needs are progressively met, with higher-order needs being particularly relevant for professional engagement and retention (Benson & Dundis, 2003). Research examining healthcare employees with Maslow’s Hierarchy of Needs found that employees wanted to feel secure, needed, and appreciated, and that considering individual needs enhanced employee motivation and commitment (Benson & Dundis, 2003). Healthcare professionals often enter their careers with strong motivations toward self-actualization through meaningful patient care, making this theoretical foundation particularly relevant for understanding professional satisfaction.

### 2.2. Herzberg’s Two-Factor Theory

Herzberg distinguishes between hygiene factors (extrinsic elements such as salary and working conditions) and motivators (intrinsic elements such as recognition and achievement). While hygiene factors prevent dissatisfaction, only motivators actively enhance satisfaction (Samira et al., 2020). This dual structure has informed many healthcare organizational strategies. In the healthcare field, particularly for rehabilitation professions, Herzberg’s Motivation-Hygiene Theory represents one of the most used frameworks for understanding job satisfaction (Tran et al., 2008). Research in healthcare laboratory settings identified health and safety, workload, salary, promotion, and recognition as key hygiene factors, while relationships with co-workers, relationships with leaders, and professional development served as primary motivators (Samira et al., 2020). However, this binary approach may not fully capture the complexity of healthcare professional experiences, particularly the critical role of interpersonal relationships and team dynamics.

### 2.3. The Mottaz Framework: A Comprehensive Approach

Mottaz defines work satisfaction “as a positive orientation toward work based upon a congruency between the worker’s perception of the work situation and his/her work values regarding those same dimensions;” it is thus an “affective response” (Mottaz, 1985, pp. 368–369). Mottaz (1985) identifies two interlinked dimensions of work satisfaction: work value and work rewards. Work value refers to the importance individuals put on each work reward. Most research on work value has compared demographic factors such as age, sex, family income, or length of time in the job position with how much people value their work (Bouwkamp-Memmer et al., 2013; Mottaz, 1985; Wang et al., 2019). Since these factors are not actionable, work value was not tabulated in this research.

Work rewards, on the other hand, present many actionable items that could be adjusted to improve job satisfaction. Mottaz (1985)’s model assesses both intrinsic and extrinsic reward variables. Intrinsic task rewards include autonomy, task significance, and task involvement. Autonomy is defined as the degree of self-direction participants feel they have while performing their job. Task significance refers to the degree to which the task is perceived as contributing to the work, while task involvement is the degree to which the task is considered interesting. These psychological constructs directly influence individual motivation and professional identity, particularly important in healthcare where professionals are motivated by patient care outcomes.

Extrinsic task rewards are either social or organizational. Supervisory assistance and colleague assistance are the two main sources of social reward. Supervisory assistance is defined by Mottaz (1985) as the degree to which workers feel supported by their direct managers, whereas colleague assistance refers to their perception of being supported in their workplace by other professionals. Other researchers have found that team dynamics (which may include colleague assistance) also influences job satisfaction (Stout et al., 2017; Vitali et al., 2020).

Organizational rewards come from having adequate working conditions, pay equity, promotional opportunities, and fringe benefits. Working conditions constitute the resources of time, space, and equipment employees need to properly execute their jobs. Poor work conditions are frequently reported as a source of job dissatisfaction (Amiri et al., 2016; Vitali et al., 2020). Healthcare providers become particularly distressed when inadequate work conditions hinder patient care (Munyewende et al., 2014). Increasing regulations, a hectic pace, long hours, and heavier workloads also contribute to decreasing job satisfaction among healthcare professionals (Mundt & Zakletskaia, 2019). Pay equity refers to the perception that one is receiving a fair salary, while promotional opportunity refers to the potential for advancement either through continuing education or direct promotion. Finally, fringe benefits such as pension plans, vacation leave, paid sick days, and medical coverage are viewed as a reward when they are appropriate to the position, while a lack of fringe benefits has a negative impact on job satisfaction (Vitali et al., 2020).

### 2.4. Gap in the Literature

Despite the extensive research on healthcare workforce challenges, significant gaps remain in our understanding of specific professional groups and practice contexts. Most studies on clinician recruitment and retention focus on physicians and nurses. While these studies produce critical insights, they cannot be fully utilized to inform recruitment and retention strategies for AHPs as they have unique professional attributes (Kueakomoldej et al., 2022).

The literature reveals a particular dearth of research examining how different models of primary care delivery influence AHP workforce experiences. Most studies treat primary care as a homogeneous setting, failing to account for the varied organizational structures, funding mechanisms, and team compositions that characterize contemporary primary care delivery. This gap is particularly relevant in Canada, where provincial variation in healthcare organization creates diverse primary care contexts that may differentially impact workforce recruitment and retention.

Furthermore, while job satisfaction is widely recognized as a critical factor in workforce sustainability, limited research has systematically examined how specific job satisfaction frameworks apply to AHPs working in primary care settings. Most of the workforce research focuses on acute care environments, leaving primary care-specific factors underexplored and potentially overlooked in workforce planning and intervention development.

### 2.5. Rationale for Framework Selection

While Herzberg’s Two-Factor Theory provides valuable insights into healthcare job satisfaction, the Mottaz framework was selected for this study due to its multidimensional and actionable structure. Unlike models that focus primarily on intrinsic versus extrinsic factors, the Mottaz (1985) model systematically also incorporates social rewards, offering a more holistic understanding of what drives satisfaction, recruitment, and retention in healthcare settings. Research in Cyprus public hospitals revealed that achievements ranked first among motivators, followed by remuneration, co-workers, and job attributes, with healthcare professionals tending to be motivated more by intrinsic factors (Lambrou et al., 2010).

The framework’s emphasis on actionable organizational factors aligns with behavioral science principles by focusing on modifiable environmental variables that influence professional behavior and career decisions. For AHPs such as respiratory therapists who work in complex interprofessional environments, the inclusion of social rewards provides critical insights into team dynamics and collaborative relationships that may not be captured by other theoretical approaches.

In the healthcare field, particularly for rehabilitation professions, both Herzberg’s Motivation-Hygiene Theory and Mottaz’s concepts of work values and work rewards represent the most used job satisfaction frameworks (Tran et al., 2008). However, Mottaz’s framework provides more comprehensive insights into actionable problems, making it particularly suitable for understanding the complex factors influencing respiratory therapist integration in primary care settings.

## 3. Materials and Methods

### 3.1. Methodology

Following the launch in 2018 of a Primary Care Recruitment and Retention campaign by Ontario’s Ministry of Health and Long-term Care, Dr. Chartrand collaborated with the Respiratory Therapy Society of Ontario (RTSO) to conduct a job satisfaction survey of RRTs in Primary Care. We changed the methodology when survey responses showed wide discrepancies in primary care employment conditions,^1^ yet just as many RRTs reported being satisfied as dissatisfied with their work (50% each). Adopting a pragmatic epistemology and realist ontology, we proposed qualitative research using a mixed inductive and deductive thematic analytic design (Fereday & Muir-Cochrane, 2006). The study was approved by the Health Research Ethics Board (HREB) of the University of Manitoba, HS22723, H2019:132. The COREQ checklist (see Supplementary Materials) was used to report the study (Tong et al., 2007).

### 3.2. Recruitment and Sampling

A purposeful sampling strategy was used to recruit participant RRTs who self-identified as working in primary care by advertising through the RTSO and the Manitoba Association of Registered Respiratory Therapists (MARRT). For qualitative descriptive studies, “sample size must be large enough to allow the unfolding of a ‘new and richly textured understanding’ of the phenomenon under study, but small enough… [for] ‘deep, case-oriented analysis’” (Sandelowski, 1995, p. 183). Data saturation indicates that sufficient participants have been obtained (Sandelowski, 1995).

### 3.3. Data Collection

The interview guide (see Supplementary Materials) was developed collaboratively by LC and SLH, with content inspired by missing information from the initial survey. Interview guide was piloted and refined accordingly. After receiving participant consent, LC conducted all the semi-structured interviews, with a total of 19 RRTs between June 2018 and December 2019. The interview guide was pilot tested by LC and SLH prior to interviews starting. At the beginning of the interview, participants were informed that Dr. Chartrand is a registered respiratory therapist that was interested in knowing more about the work conditions of RRTs in primary care setting to better understand why there were so few who decided to work in that particular setting. Interviews lasted from 45 min to 75 min in length.

There were no prior relationships with the participants prior to interviews and no one dropped out of the study. All interviews were conducted by phone and recorded on a USB recorder. Field notes were made during and after the interview. The recordings were transcribed by a professional transcriptionist and analysed using NVIVO 12 (QSR International Pty Ltd., 2018). Participants were given the choice to review transcripts and make corrections, but only one participant agreed to do so.

### 3.4. Data Analysis

The data analysis process followed a collaborative approach involving the research team. SB and OJ, both respiratory therapists with experience working in primary care settings, along with DK (research assistant) and LC, each independently coded three interviews initially. The team then convened on three separate occasions to discuss the codes that had been identified, engage in collaborative dialogue, and ensure consensus on the emerging themes and code structure.

During these consensus meetings, the team co-created inductive codes while applying the Mottaz intrinsic and extrinsic rewards framework as a deductive lens. The Mottaz framework was used to identify nodes for the deductive component, encompassing all intrinsic and extrinsic rewards described in Mottaz’s theoretical model. Simultaneously, the contexts of respiratory therapist (RRT) primary care practice were inductively coded as cases, specifically: hospital-based outpatient clinics, rostered centers, and non-rostered centers for Ontario participants; and hospital-based chronic care, hospital-based outpatient clinics, and access centers for Manitoba participants.

Once the code map was finalized and agreed upon by all team members, DK proceeded to code the remaining interviews using the established coding framework. To ensure consistency in reporting, SB authored the results section for Ontario participants, while OJ wrote the results for Manitoba participants. LC then synthesized all results together, ensuring consistency and coherence across the findings.

The analysis involved cross-referencing the identified cases (practice contexts) against the nodes (intrinsic and extrinsic rewards from Mottaz’s framework), allowing for a comprehensive examination of how workplace rewards manifest across different primary care settings for respiratory therapists. A draft copy of this article was sent to participants, to ensure the result represented their situation. Furthermore, the results of this research were presented at the 2023 CSRT conference in Charlottetown, PEI. The authors received positive feedback and there was an agreement from the community of findings. We have made all efforts to follow and achieve trustworthiness principals (Adler, 2022).

## 4. Results

We found that job satisfaction (deductive) was closely tied to the specific context (inductive) in which RRTs practiced. Therefore, we describe each work context and outline the indicators of RRT job satisfaction using Mottaz’s framework (Mottaz, 1985). Across Manitoba and Ontario, RRTs identified different ways that they are integrated into primary care. First, hospital-based outpatient clinics, second, rostered centers (e.g., community access centres or primary care teams), third, non-rostered centers (identified in Ontario but do not exist in Manitoba) and finally chronic care facilities and community care (exist in both provinces, but no participants from Ontario). While similar categories emerged in both provinces, important provincial differences in structure, support, and resources shaped RRTs’ experiences in each setting. To protect the identity of the participants (This setting is a small community of RRT and any identifier would risk compromising anonymity), we are not disclosing demographics data (even if they were captured), we only identify our participants by province, not even using pseudonyms.

### 4.1. Hospital Based Out-Patient Clinics

In both Ontario and Manitoba, some hospitals operate outpatient clinics where RRTs provide specialized care aimed at improving patient outcomes. These clinics often serve as a bridge between hospital-based acute care and community or primary care settings. While the structures and resources may differ between provinces, RRTs in both settings described their work in hospital-based outpatient clinics as highly rewarding, particularly in terms of autonomy, patient relationships, and the perceived impact of their work.

In Ontario, RRTs often work in collaboration with primary care physicians through structured programs such as asthma clinics. One RRT shared:

*“I am also involved with primary care asthma program, so we have an adult and a pediatric asthma clinic. With the adult asthma clinic, we have a half-day session that we do in hospital with a primary care physician”*.

(RRT, ON)

Similarly, in Manitoba, RRTs also collaborate with respirologists and physicians in outpatient settings. Some serve urban populations in larger centers, while others are based in rural hospitals with limited staffing and resources. These RRTs often take on additional responsibilities, such as educating physicians and conducting community outreach, in addition to their hospital duties.

Across both provinces, RRTs expressed high levels of intrinsic job satisfaction. They valued the autonomy they experienced in these roles and the ability to build long-term relationships with patients. One RRT in Manitoba emphasized the preventative and proactive nature of the work:

*“We can make a bigger impact [as] respiratory therapists”*.

(RRT, MB)

Another RRT in Ontario highlighted the personal and professional fulfillment found in this setting:

*“We actually in this department have a very satisfactory position. We have been given a lot of autonomy and valued and for the most part relationships are good…. I love what I do! I feel that I bring value and that I have a positive impact on the lives of the patients that I work with, and I love that”*.

(RRT, ON)

In terms of extrinsic rewards, the experiences were more nuanced. Ontario RRTs in outpatient clinics felt well-supported by supervisors and colleagues and appreciated working conditions that mirrored those of acute care staff, minus the burdens of shift and weekend work. However, such positions were scarce.

In Manitoba, extrinsic rewards were more variable. While RRTs enjoyed strong collegial relationships and team-based recognition, they reported fewer opportunities for professional development and advancement. This was particularly pronounced in rural settings, where RRTs often work alone and face equipment shortages and time constraints. One RRT noted:

*“We have one spirometer, so we’re at the mercy of it working every day”*.

(RRT, MB)

Another shared how ongoing advocacy was necessary to maintain adequate resources:

*“I was able to quickly rectify that, show it (spirometer) to the infection control nurse, and she’s like, ‘Oh yeah, we can’t use this anymore.’ So now I have a spirometer that at least meets conditions that I can work with”*.

(RRT, MB)

This example suggests that equipment challenges may stem less from budget constraints and more from a lack of awareness about the RRT’s role within hospital systems.

Overall, while the job satisfaction in hospital-based outpatient clinics is high across both provinces due to the meaningful nature of the work and strong interdisciplinary collaboration, there remain regional disparities in terms of staffing, equipment, and institutional support, particularly in rural Manitoba. Nonetheless, RRTs in both Ontario and Manitoba described this model of care as one of the most fulfilling contexts in which to practice.

### 4.2. Rostered Centers

In both Ontario and Manitoba, some RRTs work in rostered primary care environments, settings in which patients formally register with a physician or a health team and agree to receive their care exclusively through that team. In exchange, the healthcare team commits to providing comprehensive, continuous care. In these environments, only physicians or AHPs affiliated with the center can refer patients to an RRT. This model creates a structured and team-based approach to care that supports the integration of respiratory therapy into primary care.

RRTs in both provinces reported high intrinsic task rewards in these settings, particularly in terms of autonomy and scope of practice. In Ontario, medical directives enabled RRTs to take on responsibilities such as initiating inhaler therapy before reviewing with physicians:

*“There are some medical directives that I can work under and that’s beneficial…. There are directives to start patients on inhalers and then consult with the physician and we’ll review”*.

(RRT, ON)

Similarly, RRTs in Manitoba working at Access Centers, funded through My Health Teams, highlighted how team-based models enhanced responsiveness and collaboration:

*“The docs here will cover for each other. So, if a patient needs a prescription, I can usually find a doctor here who will write the prescription for the patient even though it’s not their primary physician. If there’s an urgent need, that’s something I can do… A lot of clinics don’t have a team like a My Health Team”*.

(RRT, MB)

Extrinsic rewards, particularly social support from the team and management, were also noted positively in both provinces. RRTs appreciated respectful work environments and good relationships with physicians and other healthcare providers. Access to appropriate equipment was generally not a major concern. However, in Manitoba, many RRTs emphasized that this was due to their own advocacy rather than systemic support.

Despite these positives, organizational challenges were apparent. In Ontario, a major concern was the prevalence of part-time positions in rostered centers. Many RRTs had to piece together multiple part-time or casual roles to maintain full-time employment: “I was basically part-time or casual status at multiple jobs and trying to fill my days” (RRT, ON). Part-time roles often lacked benefits and opportunities for advancement, leading to dissatisfaction for some.

In Manitoba, the concerns were more closely tied to human resource shortages and isolation. Several RRTs reported working alone, managing long waitlists, and feeling overwhelmed by the scope of need:

*“I’m working by myself. I don’t have another RRT to work with…. Sometimes when you have a 10-to-12-week waitlist, it can get very overwhelming. Sometimes I get bogged down by that because I feel like I’m not enough and I can’t do enough, and I can’t make a big enough of a difference. That can be hard”*.

(RRT, MB)

Additionally, some Manitoba RRTs expressed concern about the erosion of their critical care skill set, which is heavily emphasized in respiratory therapy education. Feeling undervalued by colleagues in acute care settings further compounded this dissatisfaction. One RRT noted being saddened by having “lost a skill set,” specifically the critical care skills they had learned during their respiratory therapy education (RRT, MB). However, a notable advantage in Manitoba’s Access Centers was employer-supported professional development, offering some potential for growth and promotion.

In summary, while RRTs working in rostered primary care settings in both Ontario and Manitoba experienced high intrinsic satisfaction, especially due to autonomy and meaningful patient relationships, they encountered different structural and organizational barriers. Ontario RRTs struggled with underemployment and lack of full-time opportunities, while Manitoba RRTs faced more pronounced human resource shortages, role isolation, and inconsistent professional recognition. These challenges underscore the uneven development of primary care roles for RRTs across the provinces, a point further addressed in the discussion section.

### 4.3. Chronic Care Facilities and Community Care (Only Captured in Manitoba)

RRTs working in chronic care facilities are also responsible for the respiratory needs of patients in personal care homes and community programs. Respiratory therapy duties in this primary care setting include long term oxygen therapy assessments, spirometry, pulmonary rehabilitation, amyotrophic lateral sclerosis (ALS) clinics, and educating patients to use non-invasive mechanical ventilation.

RRTs were mostly satisfied with working in these contexts and reported high intrinsic rewards, especially regarding the significance of their work. They appreciated that their colleagues relied on their expertise: “The physicians come to us for everything” (RRT, MB). This also constituted a positive extrinsic reward, specifically the social reward of interprofessional team dynamics. However, RRTs felt that they did not receive the same level of support from their colleagues working in acute care at the same hospital. The impression that other RRTs did not value chronic care management had a negative impact on job satisfaction among RRTs working in hospital-based chronic care facilities. The paucity of human resources also had a negative effect on their benefits. Although RRTs based in hospitals are guaranteed five weeks of vacation every year, those working in chronic care are not able to take off a few weeks in a row because there is insufficient coverage. On the other hand, as in Ontario, RRTs working in chronic care in Manitoba are paid the same salary as RRTs working in acute care at the same hospital, so pay rates were not a source of dissatisfaction.

RRTs working in hospital-based chronic care reported lack of supervisory assistance due to government cuts. This had a negative effect on both social and organizational rewards, specifically working conditions, because RRTs ended up performing tasks that were not necessarily part of their job description. Lack of resources made their work even more dissatisfying when it negatively impacted their ability to care for patients. Such extrinsic factors reduced their overall sense of job satisfaction.

### 4.4. Non-Rostered Centers (Only Captured in Ontario)

Most of the non-rostered centers in Ontario are Community Health Centers (CHCs). Staff physicians receive salaries rather than fees for services rendered; their wages and those of the AHPs come out of each center’s operating budget. Unlike rostered centers, non-rostered centers accept patients referred to them by any family physician, including those working in hospitals. RRTs working in these centers also see outside patients who do not have physicians tied to the clinic. As one RRT explained:

*“We’re in a unique situation where I work [in] that we have 77 primary care providers. We also see people outside that are referred from the hospitals or walk-in clinics that don’t have providers. So follow-up care is an issue. We have about a six-month waitlist to see us”*.

(RRT, ON)

Like their counterparts in rostered centers, RRTs working in non-rostered centers generally experienced positive intrinsic rewards. They also reported positive extrinsic social rewards thanks to having good relationships with their colleagues: “I have great co-workers. That’s the real reason I’m there. We really work well as a team” (RRT, ON). However, extrinsic organisational rewards were much less satisfactory. This work situation provides RRTs with a large referral pool, so they do not feel as underutilized as RRTs working in rostered centers feel. This would constitute a positive organisational reward if it were not for the problem of heavy workloads and its effect on the level of care they can provide. Patients must wait anywhere from 2 to 26 weeks to see an RRT at a non-rostered center.

Another disadvantage of working at non-rostered centers was poor working conditions. RRTs pointed out that they lacked new and up-to-date equipment. Lack of sufficient workspace was another source of dissatisfaction. RRTs complained that bathrooms, closets, or laundry rooms had been turned into tiny offices and renovated to remove carpet. Sub-optimal working conditions can also have an impact on intrinsic task rewards. RRTs may end up providing a “good enough” level of care that does not meet their own personal standards. It also makes them resent doing other tasks such as scheduling patients that could easily be performed by an administrative assistant.

Whether working in rostered or non-rostered primary care centers, RRTs had similar issues with another aspect of potential organisational reward: pay equity. RRTs working in hospital settings are paid more than those in rostered and non-rostered primary care centers, but less than other AHPs. RRTs had to advocate to raise their band (pay) level:

*“We’re band 8. That’s because of the work I [did as] a part of the RTSO and ACTO [a company that evaluates job descriptions and suggests salary ranges] within the past few years. We were on band 7 but then we did a review of the job description of an RRT in primary care. The company that was doing the review bumped us up to band 8 with all the other allied health professionals that are like what we do”*.

(RRT, ON)

Even with the increase in pay, the difference between the wages of RRTs in acute care versus those in primary care remains very large, with a maximum annual income gap of CAD $18,665.

## 5. Discussion

Our findings align with existing research on job satisfaction among AHPs, and they also offer important insights into how RRTs are integrated into diverse models of primary care delivery in Ontario and Manitoba. Amidst growing economic and societal pressures, Canadian healthcare continues to experience fragmentation across acute, primary, and community care sectors (Valaitis et al., 2020). Our study reinforces the call for more integrated models of care that improve equity and continuity of services, particularly by supporting and retaining health professionals such as RRTs across all regions and contexts.

While primary care in Canada is not standardized in terms of funding models, wages, benefits, or working conditions, our study highlights how these contextual differences significantly influence job satisfaction and workforce retention. This is particularly relevant given the strong relationship between job satisfaction and both employee retention and the quality of care delivered to patients (Halcomb et al., 2012). Consistent with earlier findings, inadequate remuneration remains a key source of dissatisfaction for RRTs, especially in Ontario’s non-rostered or part-time primary care settings (Aspden et al., 2021; Porter & Lexén, 2022).

Where this research contributes new knowledge is in demonstrating how primary care is operationalized differently across provinces and settings, and how these structural differences affect both the ability to deliver care and the overall satisfaction of RRTs. For example, Ontario has no shortage of RRT graduates, thanks to seven educational programs in the province. Yet retention is an ongoing challenge, particularly in non-rostered centers where part-time contracts lack job security, benefits, and adequate pay. These working conditions not only lead to turnover but also contribute to the devaluation of primary care roles, with acute care colleagues often perceiving primary care work as less important, which undermines intrinsic and social rewards (Cagan & Gunay, 2015; Jabbari et al., 2014). However, retention improves significantly when RRTs work in more supportive environments such as hospital-based outpatient clinics or rostered centers, where they receive fair pay, stable hours, and recognition from their teams.

In contrast, Manitoba RRTs tend to remain in primary care roles once integrated, citing both intrinsic rewards (e.g., autonomy, patient continuity) and extrinsic social rewards (e.g., respect from peers, effective team collaboration). Participants particularly valued their role in preventing emergency department visits and contributing to patient well-being through long-term relationship-building—a theme echoed in the literature linking patient-centered care with job satisfaction and improved outcomes (Stout et al., 2017). Moreover, team-based care in Manitoba’s Access Centers and My Health Teams allowed RRTs to collaborate with other providers in ways that promoted trust, communication, and shared purpose, hallmarks of effective integrated primary care teams (Mundt & Zakletskaia, 2019; Stout et al., 2017; Vitali et al., 2020).

Another benefit to primary care practice in Manitoba was work–life balance. While rural RRTs may experience limitations in vacation flexibility due to solo staffing, most reported satisfaction with their daytime schedules and absence of shift work. Manitoba RRTs also earn wages equal to or higher than their acute care counterparts, likely linked to educational qualifications. The University of Manitoba’s Bachelor of Respiratory Therapy (BRT) program prepares graduates for broader roles and higher compensation, potentially incentivizing more RRTs to remain in community settings.

However, recruitment remains a challenge in Manitoba, due to a province-wide shortage of RRTs. This affects both acute and primary care but is especially acute in rural areas, where understaffing jeopardizes service delivery. Without sufficient workforce capacity, primary care roles may be perceived as competing with hospital staffing needs. Furthermore, the lack of primary care clinical placements in RRT education programs limits students’ exposure to these roles. As noted in previous research, work-integrated learning opportunities play a critical role in motivating new graduates to pursue careers in specific areas (Wheelahan & Moodie, 2020). The current focus on urban, critical care placements contributes to a ‘culture’ that undervalues primary care, limiting the pipeline of RRTs prepared or willing to work in this field.

Taken together, our findings support the growing recognition that effective primary and community care delivery models require more than infrastructure, they depend on a well-supported, adequately compensated workforce. By addressing recruitment and retention issues, valuing interprofessional collaboration, and integrating primary care more thoroughly into RRT education and health system planning, we can improve both patient and provider outcomes. As provinces across Canada seek to reform and expand primary care services, RRTs should be recognized as key contributors to preventive, patient-centered, and cost-effective care, especially in underserved and rural communities.

### 5.1. Practical Recommendations for Healthcare Organizations and Managers

Based on our findings, several targeted interventions could improve RRT job satisfaction and workforce sustainability in primary care. These recommendations are organized by key stakeholder groups to facilitate implementation.

In Ontario, organizations should prioritize establishing province-wide minimum compensation standards and converting part-time positions to full-time roles with benefits to directly address retention barriers. Management strategies should focus on creating supportive team environments, ensuring adequate workspace and equipment, and providing clear career advancement pathways within primary care settings.

In Manitoba, organizational leaders should implement strategic workforce development initiatives, including team-based coverage models to reduce professional isolation in rural settings and systematic recruitment initiatives targeting new graduates. While the Manitoba government has increased educational seats in the BRT program, enrollment has not significantly increased, suggesting that recruitment challenges extend beyond capacity limitations to include program awareness and career pathway clarity.

### 5.2. Practical Recommendations for Educational Institutions

Respiratory therapy programs should incorporate mandatory primary care clinical placements to increase student exposure to these roles and reduce the acute care bias in professional socialization. Educational institutions should develop competency frameworks specific to primary care practice, create partnerships with primary care organizations for sustained clinical education opportunities, and design continuing education programs to support practicing RRTs transitioning from acute to primary care settings.

### 5.3. Practical Recommendations for Policymakers and Decision-Makers

Cross-provincial initiatives should focus on developing standardized medical directives for RRTs, creating interprofessional education modules to enhance role recognition, and establishing dedicated funding streams for primary care respiratory therapy services. Provincial health ministries should develop retention strategies that address jurisdiction-specific barriers, compensation and employment security in Ontario, workforce capacity and rural recruitment in Manitoba.

## 6. Conclusions

This study sheds light on the emerging role of RRTs in Canadian primary care, with a particular focus on how different models of care delivery in Ontario and Manitoba shape job satisfaction, recruitment, and retention. Our findings demonstrate that job satisfaction among RRTs is highly context-dependent and varies across provinces. In Ontario, RRTs working in hospital-based outpatient clinics and rostered centers reported greater satisfaction due to supportive environments, team integration, and improved working conditions, while those in non-rostered centers struggled with underemployment and undervaluation. In contrast, Manitoba RRTs generally reported high satisfaction across primary care contexts but faced significant recruitment challenges due to a province-wide shortage and limited educational exposure to primary care roles. These findings point to broader structural issues in how primary care is implemented and sustained across Canada.

This research makes several important contributions to job satisfaction theory and allied health workforce research. First, our study represents the first systematic application of Mottaz’s work rewards framework to respiratory therapists in primary care settings, demonstrating the framework’s utility for understanding profession-specific job satisfaction factors. Second, our findings extend job satisfaction theory by revealing how organizational context moderates the relationship between theoretical frameworks and workforce outcomes. The discovery that the same job satisfaction framework produces different actionable insights depending on provincial healthcare structures and care delivery models represents a significant theoretical advancement.

Third, our research addresses a critical gap in allied health professional workforce theory. While most job satisfaction research focuses on physicians and nurses, our study contributes to the limited theoretical understanding of how established frameworks apply to other healthcare professionals working in evolving care delivery models. Finally, our inductive discovery of distinct primary care organizational models that fundamentally shape professional experiences provides new theoretical insights into person-environment fit in healthcare settings, suggesting that context-specific factors may be more influential than previously recognized in determining job satisfaction outcomes.

Our study offers several important lessons for future workforce research design. The mixed inductive-deductive approach proved particularly valuable, allowing us to discover previously unrecognized organizational models (inductive) while applying established theoretical frameworks (deductive) to understand their impact on job satisfaction. This methodological combination enabled us to capture both emergent phenomena and theoretically grounded insights that neither approach alone could have achieved.

The cross-provincial comparative design demonstrated the importance of examining policy and organizational contexts rather than treating primary care as a homogeneous setting. Future studies should consider how different healthcare governance structures, funding mechanisms, and organizational models influence workforce experiences. Additionally, our use of semi-structured interviews revealed rich, contextual data that quantitative approaches alone might have missed, particularly the discovery of distinct care delivery models that fundamentally shaped professional experiences. However, future research would benefit from incorporating quantitative validation of qualitative findings and developing instruments to measure context-specific job satisfaction factors across different organizational models.

Despite the relevance of our findings, this study has several limitations that suggest directions for future research. Data collection occurred before the COVID-19 pandemic, which fundamentally altered primary care delivery and may have changed job satisfaction factors significantly. The two-province limitation restricts generalizability, and most participants held full-time positions, potentially underrepresenting part-time worker experiences. Future research should examine post-pandemic workforce dynamics, expand to additional provinces, and specifically target part-time and casual workers.

Research priorities should include quantitative validation of our findings through survey research, longitudinal studies examining how job satisfaction factors change over time, and effectiveness studies of different primary care delivery models on both provider satisfaction and patient outcomes. As healthcare increasingly incorporates virtual care modalities, studies should assess how these innovations affect RRT job satisfaction and role integration. Finally, continued qualitative research will be essential for understanding the evolving complexity of job satisfaction among allied health professionals as healthcare delivery models continue to transform.

As interprofessional, patient-centered care becomes increasingly central to healthcare transformation, ensuring a stable, well-supported allied health workforce will be essential for system sustainability. Our research demonstrates that effective workforce strategies must be context-specific, addressing the unique organizational, policy, and professional factors that influence job satisfaction in different healthcare environments. Strengthening the role of RRTs in primary care holds significant promise, not only for professional well-being but also for delivering high-quality, patient-centered care that meets the evolving needs of the Canadian population.

## Data Availability

Data is unavailable due to privacy or ethical restrictions.

## Note

1 For example, annual salaries ranged from CAD $35,000 to CAD $75,000 for full-time RRTs.

## Supplementary Materials

The following supporting information can be downloaded at: https://www.mdpi.com/article/10.3390/bs15101301/s1.

## Abbreviations

The following abbreviations are used in this manuscript:

RRTs	Registered Respiratory Therapists
AHPs	Allied Healthcare Professionals
