# Supplementary material for: Job Satisfaction of Registered Respiratory Therapists in Primary Care: Addressing Recruitment and Retention in Ontario and Manitoba
Source: Behav Sci (Basel). 2025 Sep 24;15(10):1301. doi: 10.3390/bs15101301 (PMC12561674; doi:10.3390/bs15101301)
Supplement: Supplementary file 1 [file behavsci-15-01301-s001.zip › behavsci-3585133-supplementary.pdf]

Section/Topic	Item No	Checklist item	Reported on page No
**Domain 1: Research team and reflexivity**			
Personal Characteristics			
Interviewer/facilitator	1	Which author/s conducted the interview or focus group?Interviewer/facilitator	6
Credentials	2	What were the researcher's credentials? E.g. PhD, MD	6
Occupation	3	What was their occupation at the time of the study?	6
Gender	4	Was the researcher male or female?	F
Experience and training	5	What experience or training did the researcher have? Relationship with participants	6
Relationship with participants			
Relationship established	6	Was a relationship established prior to study commencement?	6
Participant knowledge of the interviewer	7	What did the participants know about the researcher? e.g. personal goals, reasons for doing the research	6
Interviewer characteristics	8	What characteristics were reported about the interviewer/facilitator? e.g. Bias, assumptions, reasons and interests in the research topic	6
**Domain 2: study design**			
Theoretical framework			
Methodological orientation and Theory	9	What methodological orientation was stated to underpin the study? e.g. grounded theory, discourse analysis, ethnography, phenomenology, content analysis	5
Participant selection			
Sampling	10	How were participants selected? e.g. purposive, convenience, consecutive, snowball	5-6
Method of approach	11	How were participants approached? e.g. face-to-face, telephone, mail, email	6
Sample size	12	How many participants were in the study?	6
Non-participation	13	How many people refused to participate or dropped out? Reasons?	6
Setting of data collection	14	Where was the data collected? e.g. home, clinic, workplace	6
Presence of non-participants	15	Was anyone else present besides the participants and researchers?	6
Description of sample	16	What are the important characteristics of the sample? e.g. demographic data, date	6
Data collection			
Interview guide	17	Were questions, prompts, guides provided by the authors? Was it pilot tested?	Yes
Repeat interviews	18	Were repeat interviews carried out? If yes, how many?	no

Audio/visual recording	19	Did the research use audio or visual recording to collect the data?	6
Field notes	20	Were field notes made during and/or after the interview or focus group?	6
Duration	21	What was the duration of the interviews or focus group?	6
Data saturation	22	Was data saturation discussed?	6
Transcripts returned	23	Were transcripts returned to participants for comment and/or correction?	6
Domain 3: analysis and findingsz<br>Data analysis			
Number of data coders	24	How many data coders coded the data?	6
Description of the coding tree	25	Did authors provide a description of the coding tree?	See results
Derivation of themes	26	Were themes identified in advance or derived from the data?	6
Software	27	What software, if applicable, was used to manage the data?	6
Participant checking	28	Did participants provide feedback on the findings?	6
Reporting			
Quotations presented	29	Were participant quotations presented to illustrate the themes / findings? Was each quotation identified? e.g. participant number	yes
Data and findings consistent	30	Was there consistency between the data presented and the findings?	yes
Clarity of major themes	31	Were major themes clearly presented in the findings?	yes
Clarity of minor themes	32	Is there a description of diverse cases or discussion of minor themes?	yes

Interview Guide: Respiratory Therapists in Primary Care

Opening:

Introduce myself then …

Thank you for participating in this study. I'm interested in understanding your experiences as a respiratory therapist working in primary care. There are no right or wrong answers - I simply want to learn about your perspective and experiences.

Section 1: Present Employment and Daily Practice

1. Please describe what a typical day of work looks like for you.
   Probe: Can you walk me through from when you arrive to when you leave?
2. Do you think you could do more for your patients than what you are doing now?
   Follow-up: Do you think respiratory therapists could be used more often and more appropriately in primary care settings? Why or why not?
3. What are the main challenges of your present employment?
   Probe: Can you give me some specific examples?
4. Do you have the proper equipment to do your work?
   Follow-up: Can you tell me about any gaps in equipment or resources?
5. Do you have the proper support from your team to do your work?
   Probe: What does good team support look like to you?
6. Tell me about your involvement in care coordination or integration.
   Probe: How do you work with other healthcare providers?
7. Are you involved in any community health initiatives? (e.g., Health Links)
8. How and from whom do you receive your referrals?
   Follow-up: How does this referral process work in practice?
9. Who makes up your typical patient population? (e.g., asthma, COPD, other respiratory conditions)
10. How many patients do you typically see? Do you think this workload is appropriate?
    Probe: What would be an ideal patient load for you?

Section 2: Professional Journey

11. How long have you been a respiratory therapist, and how long have you worked at this clinic?
12. How did you learn about this opportunity?
    Follow-up: What attracted you to primary care specifically?
13. Are you aware of how long this position was vacant before you applied?
    Follow-up: What do you think might explain any vacancy period?
14. Besides your respiratory therapy licensing, what additional qualifications or training did you need for this role?

15. What challenges did you face when you first started, particularly any gaps between your formal education and the reality of this job?
    Probe: How did you address these challenges?

Section 3: Work Satisfaction and Rewards

16. Let's talk about your employment benefits. Do you receive vacation time, pension, and health benefits?
17. Are you comfortable sharing what salary band your position falls under?
18. Overall, how satisfied are you with your job and your employer?
    Probe: What contributes most to your satisfaction or dissatisfaction?
19. How would you describe your work relationships?
    Follow-up: With colleagues? With patients? With other healthcare providers?
20. Tell me about your autonomy and decision-making in this role.
    Probe: How satisfied are you with the level of independence you have?
21. What opportunities do you have for career advancement, continuing education, or professional development?
    Follow-up: How important are these opportunities to you?

Section 4: Context and Comparison

22. What makes your clinic unique compared to other clinical settings you've heard about or experienced?
23. Do you ever collaborate with respiratory therapists who work in home care or hospital settings?
    Follow-up: How does that collaboration work?
24. How would you compare your role to that of respiratory therapists in home care or hospital settings?
    Probe: What are the key differences in day-to-day responsibilities?
25. What are the main advantages and disadvantages of working in primary care as a respiratory therapist?

Closing Questions

26. What would you want other respiratory therapists to know about working in primary care?
27. If you could change one thing about your current role or primary care respiratory therapy in general, what would it be?
28. Is there anything else about your experience that you think would be important for me to understand?
